# Supplementary material for: Middle Segment-Preserving Pancreatectomy to Avoid Pancreatic Insufficiency: Individual Patient Data Analysis of All Published Cases from 2003–2021
Source: J Clin Med. 2023 Mar 3;12(5):2013. doi: 10.3390/jcm12052013 (PMC10003839; doi:10.3390/jcm12052013)
Supplement: Supplementary file 1 [file jcm-12-02013-s001.zip › MPP-IPD - Supplementary Table S1 - Extracted data.pdf]

Table S1. Individual patient data extracted from MPP case studies

Study variables	Extra Information
Study variables	**Extra Information**
First author	
Publication year	
Country of surgical center	
Patient baseline characteristics	
Age at surgery	
Sex	
ASA classification (ASA°)	If not explicitly available from primary publication/author information, independently estimated by TMP and JD on basis of provided patient data (differences resolved via consensus).
Pre-existing diabetes mellitus	Defined if patient has high blood glucose and takes anti-diabetic drugs, or as stated. Conditions such as impaired glucose tolerance due to diet or exercise was not regarded as diabetes mellitus.
Pre-existing exocrine insufficiency	Defined if patient has diarrhoea/steatorrhea, flatulence, abdominal cramps after eating.
Pancreatic head and tail pathology	Surgical indication for partial pancreatic resection and details of underlying pathologies for each end of the pancreas
Number of lesions in the pancreatic head	As diagnosed by preoperative radiology and confirmed by postoperative histopathology
Number of lesions in the pancreatic body and tail	As diagnosed by preoperative radiology and confirmed by postoperative histopathology
Surgical procedures	
Proximal operation	Surgical techniques used at the proximal site
Distal operation	Surgical techniques used at the distal site
Anastomosis method	Pancreatic anastomosis techniques
Transection method	Transection and closure methods for distal pancreatic parenchyma
Intra-operative outcomes	
Operation time (min)	
Blood loss (mL)	
Length of the pancreatic remnant (cm)	
Volume of the pancreatic remnant (% of original)	
Post-operative course and complications	
Dignity of (peri-) pancreatic disease	As classified by final histopathological diagnosis
Weight loss (kg)	
Weight loss (% of original)	
Uneventful post-operative stay (present/absent)	Refers to initial post-operative hospital stay and not long-term events
Any noted complication (present/absent)	Classified into binary variables for: morbidity, post-operative pancreatic fistula (POPF; additionally classified according to ISGPF °B-C)[1,2], delayed gastric emptying (DGE; additionally classified according to ISGPS °A-C)[3] and Other (complication described with text)
Readmission to hospital (present/absent)	
Post-operative diabetes mellitus (present/absent)	Defined if patient has high blood glucose and takes anti-diabetic drugs, or as stated.
Endocrine insufficiency (present/absent)	
Post-operative exocrine insufficiency (present/absent)	
Post-operative hypoglycaemic event (present/absent)	
Length of stay in hospital (days)	
Post-operative diabetic therapy	None, Diet, OAD or Insulin
Post-operative insulin dosage (U/day)	
Post-operative HbA1c (%)	
Post-operative FBG (mmol/L)	
Post-operative OGTT (mmol/L)	
Enzyme supplements (present/absent)	
Enzyme suppl. lipase dosage (U/day)	
Survival information	
Overall survival/follow-up (months)	
Disease-free survival (months)	
Progression-free survival (months)	
Death event during follow-up	
Cause of death	
Recurrence of malignancy (present/absent)	

OAD: oral antidiabetic drugs (non-insulin) including biguanides, sulfonylureas, meglitinide, thiazolidinedione, dipepdtidyl, peptidase 4 inhibitors, sodium-glucose cotransporter inhibitors and α-glucosidase inhibitor

References

1. Malleo, G.; Pulvirenti, A.; Marchegiani, G.; Butturini, G.; Salvia, R.; Bassi, C. Diagnosis and management of postoperative pancreatic fistula. *Langenbecks Arch Surg* **2014**, *399*, 801–810, doi:10.1007/s00423-014-1242-2.
2. Bassi, C.; Marchegiani, G.; Dervenis, C.; Sarr, M.; Abu Hilal, M.; Adham, M.; Allen, P.; Andersson, R.; Asbun, H.J.; Besselink, M.G.; et al. The 2016 update of the International Study Group (ISGPS) definition and grading of postoperative pancreatic fistula: 11 Years After. *Surgery* **2017**, *161*, 584–591, doi:10.1016/j.surg.2016.11.014.
3. Wente, M.N.; Bassi, C.; Dervenis, C.; Fingerhut, A.; Gouma, D.J.; Izbicki, J.R.; Neoptolemos, J.P.; Padbury, R.T.; Sarr, M.G.; Traverso, L.W.; et al. Delayed gastric emptying (DGE) after pancreatic surgery: A suggested definition by the International Study Group of Pancreatic Surgery (ISGPS). *Surgery* **2007**, *142*, 761–768, doi:10.1016/j.surg.2007.05.005.
