# Supplementary material for: Middle Segment-Preserving Pancreatectomy to Avoid Pancreatic Insufficiency: Individual Patient Data Analysis of All Published Cases from 2003–2021
Source: J Clin Med. 2023 Mar 3;12(5):2013. doi: 10.3390/jcm12052013 (PMC10003839; doi:10.3390/jcm12052013)
Supplement: Supplementary file 1 [file jcm-12-02013-s001.zip › MPP-IPD - Supplementary Tables - IPD.pdf]

Table S2. Individual baseline characteristics for MPP group.

Patient	Study	Year	Country	Age	Sex	ASA	DM	Pathology (Head)	Pathology (Tail)	# Lesions (Head)	# Lesions (Tail)	Indication	Dignity
1	1	2003	USA	31	f	1	0	SPN	SPN	1	1	Neoplasia	Benign
2	2	2007	Japan	66	m	3	DM	AC	IPMN	1	1	Synchronous	Malignant
3	3	2009	Italy	28	m	2	0	PNEN	PNEN	2	1	Neoplasia	Benign
4	3	2009	Italy	32	m	2	0	PNEN	PNEN	1	1	Neoplasia	Malignant
5	3	2009	Italy	70	m	2	0	BD-IPMN	BD-IPMN	1	4	Neoplasia	Benign
6	3	2009	Italy	35	f	2	0	BD-IPMN	CP	1	0	Synchronous	Benign
7	3	2009	Italy	60	f	2	0	Retention Cyst	CP	1	0	Synchronous	Benign
8	4	2010	Japan	65	f	3	0	mRCC	mRCC	1	3	Metastatic	Malignant
9	5	2010	Japan	67	f	2.5	0	mRCC	mRCC	1	4	Metastatic	Malignant
10	6	2010	Italy	59	m	2	0	MT-IPMN	CP	1	1	Synchronous	Benign
11	7	2011	China	63	f	3	0	AC	SPN	1	1	Synchronous	Malignant
12	8	2011	Japan	69	m	2	0	BD-IPMN	BD-IPMN	1	1	Neoplasia	Malignant
13	8	2011	Japan	67	f	2	0	PNEN	PNEN	3	2	Neoplasia	Malignant
14	8	2011	Japan	69	m	2	DM	BD-IPMN	BD-IPMN	1	1	Neoplasia	Malignant
15	8	2011	Japan	83	m	3	0	dBDC	BD-IPMN	1	1	Synchronous	Malignant
16	9	2011	Japan	73	f	3	0	CC	PNEN	1	1	Synchronous	Malignant
17	10	2014	Japan	80	f	3	0	dBDC	MCA	1	2	Synchronous	Malignant
18	11	2014	Japan	76	m	2	0	BD-IPMN	MD-IPMN	1	1	Neoplasia	Benign
19	12	2014	Japan	67	m	3	DM	mRC	mRC	1	1	Metastatic	Malignant
20	13	2014	Japan	70	m	2	0	PNEN	PNEN	1	2	Neoplasia	Benign
21	14	2016	China	37	m	2	DM	CP	CP	NA	NA	Non-Neoplastic	Benign
22	14	2016	China	81	f	2	0	mRCC	mRCC	1	1	Metastatic	Malignant
23	14	2016	China	48	m	2	0	mDFSP	mDFSP	1	1	Metastatic	Malignant
24	14	2016	China	52	f	2	0	mPCC	mPCC	1	1	Metastatic	Malignant
25	14	2016	China	50	f	2	0	PNEN	PNEN	1	1	Neoplasia	Malignant
26	15	2020	France	28	f	2.5	0	PNEN	PNEN	multifocal	multifocal	Neoplasia	Malignant
27	16	2020	Japan	77	f	2.5	0	PDAC	PDAC	1	1	Neoplasia	Malignant
28	17	2021	Japan	79	m	2	NIDDM	PDAC	MT-IPMN	1	1	Synchronous	Malignant
29	18	2020	Germany	57	f	3	0	PNEN	CP	1	1	Synchronous	Benign

AC: ampullary carcinoma. ASA: American Society of Anesthesiologists classification. BD-IPMN: branch-duct intraductal papillary mucinous neoplasm. CC: colon cancer. CP: chronic pancreatitis. dBDC: distal bile duct carcinoma. DM: pre-existing diabetes mellitus. IPMN: intraductal papillary mucinous neoplasm. MCA: mucinous cystadenoma. mDFSP: metastatic dermatofibrosarcoma protuberans. MD-IPMN: main-duct intraductal papillary mucinous neoplasm. mPCC: metastatic pheochromocytoma. mRC: metastatic rectal cancer. mRCC: metastatic renal cell carcinoma. MT-IPMN: mixed-type intraductal papillary mucinous neoplasm. NIDDM: non-insulin dependent diabetes mellitus. PDAC: pancreatic ductal adenocarcinoma. PNEN: pancreatic neuroendocrine neoplasia. SPN: solid pseudopapillary neoplasm.

Table S3. Individual baseline characteristics for TP group.

Patient	Year	Country	Age	Sex	ASA	DM	Pathology (Head)	Pathology (Tail)	Indication	Dignity
30	2003	Germany	37	f	2	0	SCA	PNEN	Synchronous	Benign
31	2006	Germany	51	f	2	0	CP	CP	Non-Neoplastic	Benign
32	2009	Germany	66	m	1	0	CP	CP	Non-Neoplastic	Benign
33	2009	Germany	66	f	2	0	BD-IPMN	BD-IPMN	Neoplasia	Benign
34	2010	Germany	63	m	2	NIDDM	MT-IPMN	MT-IPMN	Neoplasia	Benign
35	2011	Germany	77	f	3	0	PNEN	MT-IPMN	Synchronous	Benign
36	2011	Germany	65	f	2	0	MT-IPMN	MT-IPMN	Neoplasia	Benign
37	2013	Germany	56	m	4	0	CP	CP	Non-Neoplastic	Benign
38	2014	Germany	63	f	2	0	MT-IPMN	MD-IPMN	Neoplasia	Benign
39	2014	Germany	70	f	2	NIDDM	MT-IPMN	MT-IPMN	Neoplasia	Benign
40	2014	Germany	58	m	3	0	BD-IPMN	PNEN	Synchronous	Benign
41	2016	Germany	72	m	3	0	MT-IPMN	MD-IPMN	Neoplasia	Benign
42	2017	Germany	75	f	3	0	MT-IPMN	MT-IPMN	Neoplasia	Benign
43	2019	Germany	64	f	2	NIDDM	MT-IPMN	MT-IPMN	Neoplasia	Benign

ASA: American Society of Anesthesiologists classification. BD-IPMN: branch-duct intraductal papillary mucinous neoplasm. CP: chronic pancreatitis. DM: pre-existing diabetes mellitus. MD-IPMN: main-duct intraductal papillary mucinous neoplasm. MT-IPMN: mixed-type intraductal papillary mucinous neoplasm. NIDDM: non-insulin dependent diabetes mellitus. PNEN: pancreatic neuroendocrine neoplasia. SCA: serous cystic adenoma.

Table S4. Individual patient data for surgical procedures and intraoperative outcomes in MPP patients.

Patient	Study	Proximal Operation	Distal Operation	Additional Operation Details	Anastomosis	Transection	Operation Time (min)	Blood Loss (mL)	Remnant Length (cm)	Remnant Volume (% original)
1	1	PPPD	DP		NA	Stapler	270	800	2.00	15.0
2	2	PPPD	DP		PJ	Stapler	670	1729	6.00	NA
3	3	PPPD	DP		PJ	Scalpel + sutures	330	150	7.00	NA
4	3	PPPD	DP		PJ	Scalpel + sutures	360	200	8.50	NA
5	3	PPPD	DP		PJ	Scalpel + sutures	420	300	7.50	NA
6	3	PPPD	DP		PJ	Scalpel + sutures	365	150	9.00	NA
7	3	PD	DP		PJ	Scalpel + sutures	440	200	6.00	NA
8	4	UPR	DP		None	Scalpel + sutures	440	1720	5.00	40.0
9	5	SSPPD	DP		PJ	Stapler	475	740	7.20	20.0
10	6	PPPD	SPDP		PJ	Scalpel + sutures	330	250	5.00	27.5
11	7	PD	DP		NA	NA	450	400	7.00	40.0
12	8	IPHR	DP		NA	NA	368	900	NA	NA
13	8	DPPHR	SPDP	+ right lobectomy of the liver	PJ	Scalpel + sutures	643	1020	5.00	NA
14	8	IPHR	SPDP		PJ	Scalpel + sutures	540	1420	NA	NA
15	8	SSPPD	SPDP		PJ	Scalpel + sutures	447	1730	7.00	NA
16	9	PD	SPDP	+ right hemicolectomy	PJ	Scalpel + sutures	406	960	6.00	NA
17	10	PD	DP	converted SSPPD to conventional PD, with ’reversed’ gastrojejunostomy and Braun enteroenteric anastomosis	reversed PG	Scalpel + sutures	552	5055	5.15	37.4
18	11	SSPPD	DP		PJ	Scalpel + sutures	415	655	8.00	56.9
19	12	SSPPD	DP		PJ	Stapler	669	770	4.10	33.0
20	13	SSPPD	DP	Laparoscopic	PJ	Scalpel + sutures	594	534	5.00	23.8
21	14	DPPHR	SPDP	Beger’s procedure	PJ	NA	370	1200	4.61	18.4
22	14	PPPD	DP		PJ	NA	250	400	5.18	39.5
23	14	PPPD	DP	+ resection for extrapancreatic lesions of mDFSP (metachronously, synchronously)	PJ	NA	285	600	3.21	38.9
24	14	PPPD	DP	+ resection for extrapancreatic lesions of mDFSP (metachronously, synchronously)	PJ	NA	615	5500	4.89	35.8
25	14	PPPD	SPDP		PJ	NA	330	800	5.54	18.2
26	15	PD	DP	+ left hepatectomy	PJ	Scalpel + sutures	660	500	5.00	18.0
27	16	PD	DP	+ SMV resection and reconstruction	PJ	NA	590	1530	4.00	34.6
28	17	PD	DP		PJ	Stapler	490	1440	4.00	38.9
29	18	PPPD	SPDP		PJ	Stapler	321	900	4.50	25.0

ASA: American Society of Anesthesiologists classification. DP: distal pancreatectomy with splenectomy. DPPHR: duodenum preserving pancreatic head resection (Beger, Bern, Frey). IPHR: inferior pancreatic head resection. PD: pancreaticoduodenectomy (Kausch-Whipple). PG: pancreaticogastrostomy. PJ: pancreaticojejunostomy. PPPD: pylorus preserving pancreaticoduodenectomy (Traverso-Longmire). SMV: superior mesenteric vein. SPDP: spleen preserving distal pancreatectomy. SSPPD: subtotal stomach preserving pancreaticoduodenectomy. UPR: uncinate process resection. Procedures prefixed by '+' represent additional procedures performed.

Table S5. Individual patient data for surgical procedures and intraoperative outcomes in TP patients, showing also justification for patient selection

Patient	Operation	Assessed potential for MPP as estimated length of unaffected corpus-parenchyma in cm (summarised evidence)*	Operation Time (min)	Blood Loss (mL)
30	PP-SP-TPD	4-5cm unaffected corpus-parenchyma (Final histology: 4.5cm head section included 2cm SCA, 5cm tail section included 1cm NET)	350	NA
31	TPD	5cm unaffected corpus-parenchyma (Preoperative: suspected multilocular MD-IPMN up to max. 4cm; Intraoperative: palpation of tumor in pancreatic-head; Final histology: not confirmed, only sectional-CP)	277	1200
32	PP-TPD	>3cm unaffected corpus-parenchyma (Preoperative: suspected multilocular IPMN at least in pancreatic-head and -tail; Final histology: not confirmed, only CP with pseudocysts and fibrosis especially in -head and -tail)	248	600
33	PP-SP-TPD	Completely unaffected corpus-parenchyma (Preoperative: suspected MT-IPMN in pancreatic-head and -tail. Final histology: BL-BD-IPMN <1.3cm with normal main duct only at the resection margin with accentuated obstructed and fibrotic parenchyma)	341	900
34	PP-TPD	>9cm unaffected corpus-parenchyma (Final histology: 13cm head section included 3cm MT-Cis-IPMN, 6.5cm tail section included 1.2cm MT-Cis-IPMN, lesion free resection margins)	235	500
35	PP-TPD	Almost completely unaffected corpus-parenchyma (Preoperative: MRI/CT with suspected multilocular IPMN in pancreatic-head and progredient in proximal -tail; Final histology: 14cm total resection included 8mm benign neck-NET + 1cm benign tail-MT-IPMN + 1.5cm benign tail-MT-IPMN)	260	600
36	PP-TPD	>5cm unaffected corpus-parenchyma (Final histology: 13cm head section included 1.2cm/1cm head/uncinate process-MT-Cis-IPMN, 6cm tail section included 1cm MT-Cis-IPMN)	225	600
37	TPD	Completely unaffected corpus-parenchyma (Preoperative: suspected head-IPMN and tail-IPMN; Intraoperative: suspected CP. Final histology: no IPMN and only accentuated fibrosis in -head and -tail)	215	1200
38	PP-SP-TPD	>4cm unaffected corpus-parenchyma (Preoperative radiology and final histology: 2.8cm neck-MT-IPMN adenoma + 1cm tail-MT-IPMN adenoma, >4cm free parenchyma in between the lesions)	208	700
39	TPD	>3cm unaffected corpus-parenchyma (Preoperative radiology and final histology: 2.5cm head-MT-IPMN adenoma + 1.5cm tail-MT-IPMN adenoma)	353	NA
40	TPD	Completely unaffected corpus-parenchyma (Final histology: 2.5cm head-BL-BD-IPMN + 3.5cm haemorrhage adjacent to the papilla + 1.8cm tail-pT1NETl, suspected MD-IPMN in frozen section but not confirmed at final histology, lesion free resection margins)	232	2200
41	PP-TPD	>5cm unaffected corpus-parenchyma (Final histology: 1.2cm head-BL-BD-IPMN + 1.6cm corpus-/tail- BL-BD-IPMN, suspected MD-IPMN in frozen section but not confirmed at final histology)	283	900
42	PP-SP-TPD	>3cm unaffected corpus-parenchyma (Final histology: 1.3cm head-BL-MT-IPMN + 2.5cm corpus/-tail-BL-MT-IPMN)	314	350
43	PP-SP-TPD	>3cm unaffected corpus-parenchyma (Final histology: only 1.5cm head-MT-HG/Cis-IPMN, lesion free resection margins)	368	NA

* Separately and independently evaluated by TMP and JD from summarized information of preoperative radiology, intraoperative surgical evaluation, and postoperative histopathology. For cases of divergent evaluation a consensus was found by discussion.

ASA: American Society of Anesthesiologists classification. BD: branch-duct. BL: borderline. Cis: carcinoma in situ. CP: chronic pancreatitis. HG: high-grade. IPMN: intraductal papillary mucinous neoplasm. MD: main-duct. MT: mixed-type. PP-TPD: pylorus preserving total pancreaticoduodenectomy. SP-TPD: spleen preserving total pancreaticoduodenectomy. TPD: total pancreaticoduodenectomy.

Table S6. Individual patient data for the post-operative course of MPP patients.

Patient	Study	LOS	Uneventful post-op stay	Morbidity	Readmission	POPF	DGE	Endocrine insufficiency	New-onset DM	Exocrine insufficiency (presentation)	HbA1c	FBG	OGTT	Hyp. events	Enzyme supplements (lipase dosage)	Other complications
1	1	5	1	1	1	NA	NA	0	0	0				0		Long-term symptomatic pseudocyst requiring percutaneous drainage and endoscopic transgastral stenting 1.5 months after MPP
2	2	69	0	1	0	1	0		NA	0					0	Respiratory failure
3	3	13	0	1	0	1	0	0	0	1 (Steatorrhea)			7.7		1 (60k)	
4	3	10	1	0	0	0	0	0	0	0			7.7		0	
5	3	7	1	0	0	0	0	1	IDDM	1 (Steatorrhea)	7.4			1	1 (NA)	
6	3	8	1	0	0	0	0	0	0	0			7.7		0	
7	3	16	1	0	0	0	0	1	IDDM	1 (Steatorrhea)	6.7			0	1 (NA)	
8	4	65	0	1	0	1	0	0	0	0	4.9		7.7		0	
9	5	49	0	1	0	0	0	1	IDDM	0	6.3				0	Reoperation for successful hemostasis due to early post-pancreatectomy hemorrhage (Type B PPH) from the pancreatic remnant
10	6	21	0	1	0	0	0	1	IDDM	1 (Reduced fecal elastase)					1 (NA)	Blood transfusion: peritoneal bleeding, and splenic hematoma
11	7	12	1	0	0	0	0	0	IGT	0					0	Recurrent mild attacks of acute pancreatitis after surgery (not counted for analysis because no related procedures occurred)
12	8	25	0	1	0	1	0	0	0	0					0	
13	8	139	0	1	0	1	0	0	0	0					0	
14	8	47	0	1	0	1	0	1	NA	0						
15	8	30	1	0	0	0	0	0	0	0					0	
16	9	32	0	1	0	1	0	0	0	0	5				0	
17	10	50	0	1	0	1	0	0	0	0	6	5.5			0	
18	11	21	1	0	0	0	0	0	0	0	8.1				0	
19	12	63	0	1	0	1	0	NA	NA	NA						
20	13	37	0	1	0	1	0	1	NIDDM	0					0	Cholangitis
21	14	21	0	1	1	1	0	1	NA	1 (Steatorrhea)		8.2	21.9	0	1 (60k)	Liver abscess; cured by CT-guided percutaneous drainage 1 month after MPP
22	14	52	0	1	0	1	1	1	IDDM	1 (Steatorrhea)		7.4	13.38	0	1 (60k)	
23	14	21	0	1	0	0	1	1	IGT	0		5.4	9.65		0	
24	14	60	0	1	0	0	0	0	0	0		5.5	4.01		0	Pleural effusion

Patient	Study	LOS	Uneventful post-op stay	Morbidity	Readmission	POPF	DGE	Endocrine insufficiency	New-onset DM	Exocrine insufficiency (presentation)	HbA1c	FBG	OGTT	Hyp. events	Enzyme supplements (lipase dosage)	Other complications
25	14	23	0	1	0	0	1	1	IFG	1 (Steatorrhea)		6.3	5.75		1 (30,000)	
26	15	22	1	1	0	0	0	0	0	0					1 (NA)	
27	16	96	0	1	0	1	0	1	NIDDM	0	5.3				1 (NA)	
28	17	32	0	1	1	1	0	1	NIDDM to IDDM	0	6.3			0	1 (30,000)	Cholangitis; cured conservatively by antibiotic therapy 2 months after surgery
29	18	30	0	1	0	1	1	0	0	1 (Steatorrhea)				0	1 (75,000)	Superficial wound healing disorder

DGE: delayed gastric emptying. DM: diabetes mellitus. FBG: fasting blood glucose. OGTT: oral glucose tolerance test. Hyp: hypoglycaemic. IDDM: insulin-dependent diabetes mellitus. IFG: impaired fasting glucose. IGT: impaired glucose tolerance. LOS: post-operative length of stay (days). NIDDM: non-insulin dependent diabetes mellitus. POPF: post-operative pancreatic fistula.

Table S7. Individual patient data for the post-operative course of TP patients.

Patient	Study	LOS	Uneventful post-op stay	Morbidity	Readmission	POPF	DGE	Endocrine insufficiency	New-onset DM	Exocrine insufficiency (presentation)	HbA1c	FBG	Hyp. events	Enzyme supplements (lipase dosage)	Other complications
30	13	1	0	0	0	0	1	0	1	1 (Steatorrhea, meterorism, flatulence)	9	115	1	1 (200k)	
31	28	0	1	0	0	0	1	0	1	1 (Meteroism, flatulence)	8.7	155	1	1 (400k)	Chyle leak grade A
32	17	0	1	0	0	0	1	0	1	1 (Meteroism, flatulence)	7	125	1	1 (120k)	Hypoglycemic episodes, urinary tract infection
33	24	0	1	0	0	0	1	0	1	0	5.4	110		1 (60k)	Pulmonal arterial embolism
34	11	1	0	0	0	0	1	1	NA	1 (Abnormal stools without high dose enzyme supplement)	6.7			1 (240k)	
35	42	0	1	0	0	0	1	0	1	1 (Nausea and vomiting)	6.2			1 (150k)	Chyle leak grade B, urinary tract infection, superficial wound infection
36	5	0	1	0	0	0	1	0	1	1 (Abnormal stools without high dose enzyme supplement)	6.4	160	1	1 (375k)	Gastric ischemia and colon perforation with peritonitis, hemorrhage gastrojejunostomy and branch of renal artery, pulmonal arterial empbolism
37	22	0	1	0	0	0	1	0	1	1 (Steatorrhea, nausea and vomiting)	7.5		1	1 (240k)	Pneumonia
38	18	0	1	0	0	0	1	0	1	1 (Steatorrhea, flatulence)	8.2	130	1	1 (320k)	Diabetic decompensation
39	20	0	1	0	0	0	1	1	NA	1 (Nausea)	9		1	1 (75k)	Cholangitis, superficial wound infection
40	17	1	0	0	0	0	1	0	1	1 (Abnormal stools without high dose enzyme supplement)				1 (120k)	
41	15	0	1	0	0	0	1	0	1	1 (Steatorrhea, meteorism)	6.9	140	1	1 (225k)	New-onset atrial fibrillation
42	15	0	1	0	0	0	1	0	1	1 (Steatorrhea, meteroism, flatulence)	6.7	200	1	1 (675k)	Superficial wound infection
43	13	0	1	0	0	0	1	1	NA	1 (Steatorrhea, meteroism, flatulence)	6.5	105	1	1 (12k)	Superficial wound infection

DGE: delayed gastric emptying. DM: diabetes mellitus. FBG: fasting blood glucose. Hyp: hypoglycaemic. LOS: post-operative length of stay (days). POPF: post-operative pancreatic fistula
